# Aortic and Internal Carotid Atherosclerosis in Patients with Carotid Stenosis: Semiautomatic Volumetric Analysis of Low-Attenuation Plaque on Curved Planar Reformations Using MDCT Angiographic Data

**DOI:** 10.1155/2019/5817534

**Published:** 2019-04-22

**Authors:** Hiroshi Manaka, Izumi Torimoto, Zenjiro Sekikawa, Keiichiro Kasama, Tetsuya Yamamoto, Shigeo Takebayashi

**Affiliations:** ^1^Neurosurgery, Yokohama City University Medical Center, 4-57 Urafune-cho, Minami-ku, Yokohama 232-0024, Japan; ^2^Diagnostic Radiology, Yokohama City University Medical Center, 4-57 Urafune-cho, Minami-ku, Yokohama 232-0024, Japan; ^3^Cardiovascular Surgery, Yokohama Municipal Citizens Hospital, Japan; ^4^Neurosurgery, Yokohama City University, Postgraduate School of Medicine, Fukuura, Kanazawa-ku, Yokohama, Japan

## Abstract

This retrospective study included 65 patients who underwent multidetector computed tomography (MDCT) carotid angiography; 28 patients were <70 years old (group 1), and 37 were ≥70 years old (group 2). Each low-attenuation (<30 Hounsfield units [HU]) plaque volume (LPV) and total uncalcified plaque volume ([TUPV] ≤150 HU) were semiautomatically measured on each aortic arch and internal carotid artery (ICA) curved planar reformations (CPR), using MDCT angiographic data. Correlation coefficients were employed to assess the impact of each plaque volume on various factors including ICA stenosis. The correlations (r > 0.5) were observed between aortic LPV and each ICA stenosis ratio and >30% stenosis in group 1, between aortic TUPV and male gender in group 1, and between ICA-TUPV and each aortic TUPV or the largest plaque thickness in group 2. Marginal correlations were observed between hyperlipidemia and aortic LPV and ICA-TUPV in group 1. There was no association between cerebral infarction and the aortic and ICA plaques. Both the aortic arch and ICA plaque volumes can be measured clinically. The increasing aortic LPV may be a significant factor associated with the development of ICA stenosis in patients younger than 70 years old.

## 1. Introduction

Atherosclerosis is a diffuse pathological process characterized by the deposition of lipid and other blood-borne material within the arterial wall of almost all vascular territories. Aortic arch atheromatous plaques may play a larger role in stroke risk, especially in stroke patients with undetermined etiology [1, 2]. The relation of aortic plaques to stroke mainly originates from studies with transesophageal echocardiography (TEE) [3] and the association between aortic plaques and ischemic stroke is particularly strong when the plaques are >4 mm thickness [4], although computed tomography (CT) angiography has been shown to be a higher-sensitivity and specificity tool for the detection of atherosclerotic aortic plaques than TEE [5]. Cui et al. [1], however, reported that plaques of >4 mm thickness in the aortic arch have no association with carotid plaques or intracranial arterial stenosis. In addition, Walker et al. [6] reported that analysis of internal carotid artery (ICA) atherosclerosis plaque attenuation with single-slice spiral CT does not give useful information about plaque composition.

Because of advances in multidetector computed tomography (MDCT) technology, more than 64 rows of scanning enable faster acquisition of volumetric image data with superior z-axis resolution with a thin collimation for CT angiography. Compared to complicated and time-consuming manual volumetric assessment as the plotting contour method, automatic or semiautomatic segmentation using various postprocessing techniques can greatly reduce the required time and increase the potential accuracy of measurement [7]. Recently, we found that curved planar reformations (CPR), found by using MDCT angiographic data, which allow the demonstration of plaques with attenuation-dependent color codes, can be applied to the measurement of plaque volume [8]. The purpose in this retrospective study was to establish the clinical significance of low-attenuation plaque volumetric data in aortic and internal carotid artery (ICA) atherosclerosis in patients with carotid stenosis, hyperlipidemia, and cerebral infarction.

## 2. Material and Methods

### 2.1. Study Group

Approval for this retrospective study was obtained from our institutional review board and informed consent was waived. The clinical and radiological database sets at our hospital were searched for 76 consecutive patients for whom MDCT carotid angiography for the evaluation of the internal carotid after ultrasound screening for asymptomatic atherosclerotic carotid stenosis was conducted between September 2015 and January 2018. From their clinical charts, we collected data for age, gender, hyperlipidemia (treatment or high fasting cholesterol levels), and cerebral infarction by reviewing the brain MRI reports.

### 2.2. Volumetric Data Acquisition in MDCT Carotid Angiography

The volumetric data with 0.5 mm thickness were acquired by using a 160-row MDCT unit (Aquilion Premium; Toshiba Medical Systems, Ohtawara), after an intravenous bolus injection of 420-480 mgI/kg contrast material (300 or 370 mgI iopamidol) followed by 20 mL of saline at a rate of 3.5-4.5 mL/second. The data from the aortic arch to the brain parietal lobe were acquired by a 160-row scan (160 mm x 0.5 mm collimation, a beam pitch of 0.637, a rotation speed of 0.5 second, and a table speed of 139 mm per rotation and voltage of 120 kVp). A neurosurgeon reviewed ICAs on maximum intensity projection algorithm in MDCT angiography. With the North American Symptomatic Carotid Endarterectomy Trial (NASCET) criteria, the percentage stenosis ratio between the residual luminal surface (inner-to-inner lumen) at the stenosis and the surface of the distal normal lumen (inner-to-inner lumen), where there is no stenosis, was calculated [9].

### 2.3. Curved Planar Reformations

The measurements of plaque volumes in the aortic arch were performed according to the aforementioned technique [8]. The postprocessing software that we used was a commercially available application, Aortic CPR Analyzer or CPR Analyzer (Synapse Vincent [Synapse 3D], Fuji-Film Medical Co., Tokyo, Japan).

**Figure 1:**
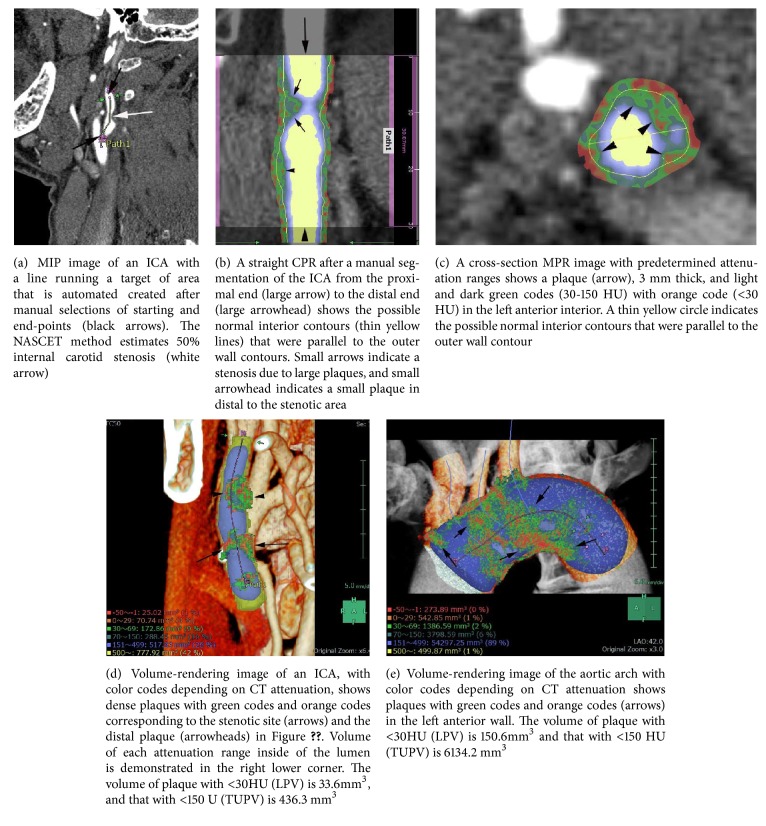
Semiautomatic volumetric data of ICA plaques with attenuation-dependent color codes in a 64-year-old man with an old cerebral infarction.

The software was used in a picture-archiving and communication system (Synapse, ver. 3.1, Fuji-Film Medical Co.). By Aortic CPR Analyzer, automated creation of the aortic arch images included three-dimensional (3-D) volume-rendering images, both straight and stretch CPR images as well as the cross-section multiplanar reconstruction (MPR) images. By CPR Analyzer, those images of the ICA were obtained after manual selection of the target ICA in 3 cm length on the maximum intensity projection (MIP) image (Figure 1(a)). The possible normal interior contours that were parallel to the outer wall contours were automatically displayed in the CPR and cross-sectional MPR images (Figures 1(b) and 1(c)). The software also allowed automated quantitative measurements of discriminative color codes based on CT attenuation in the normal interior insides with predetermined attenuation ranges in Hounsfield units (HU): −50 ~ −1 HU, 0 ~ 29 HU, 30 ~ 69 HU, 70 ~ 150 HU, and 151 ~ 500 HU [10]. Automated measurement of each area and volumes of those attenuation ranges inside the circle indicating the normal interior of the aorta or ICA were shown on the cross-section MPR segmented volume-rendering image (Figure 1(c)). The volumetric data of each attenuation code portion in the plaques were automatically displayed on the volume-rendering image after a manual segmentation of the aortic arch or target ICA on the CPR images (Figures 1(d) and 1(e)). Time required for plaque volumetric analysis in each aortic arch and an ICA plaque was 7 minutes including the time of the creation of the CPR.

User 1 undertook plaque volumetric analysis in the aortic arch. The aortic arch, which was located from proximal to the brachiocephalic arterial orifice to the horizontal plane distal to the orifice of the left subclavian artery, was manually selected on the CPR image with a reference to the volume-rendering image. The user noted that the volume of low-attenuation plaque was <30 HU and that the total noncalcified plaque was ≤150 HU in the aortic arch segment in all 65 patients. User 2 measured plaque volumes in the ICA. One radiologist reviewed multiple cross-section MPR images of each ICA and measured the largest plaque thickness with the electric caliper provided in CPR. For the evaluation of interobserver variability in ICA plaque volumetric analysis, user 3 measured the LPV in 60 ICAs in a random group of 30 patients. Furthermore, user 2 repeated the measurement of each LPV in the 60 ICAs after a 2-month interval to assess intraobserver variability. All of the reviewers and users for all of the postprocessing techniques were blinded to the patients' histories and outcomes during their independent quantitative analysis and technique evaluation.

### 2.4. Statistical Analysis

Statistical analyses were performed using Excel add-on software, XLSTAT (Addinsoft, Cologne, Germany), in each patient's group. Regarding the demographic factors, the categorical variables were compared with Pearson's chi-square test between two patients groups and the Mann-Whitney U test was used to compare the continuous variables. Mean values of LPV and TUPV and the mean ratio of LPV to TUPV in the aortic arch and ICA were also compared using the Mann-Whitney U test. A p value of 0.05 by a two-tailed test was considered statistically significant. Correlation coefficients in Spearman's rank test were employed to assess the impact of TUPV and LPV in the aortic arch and ICA on gender, cerebral infarction, hyperlipidemia, the largest plaque thickness, and ICA stenosis (% stenosis and >30% stenosis) when we used the means of the right and left carotid estimates. The correlation coefficients, each greater than 0.5 and 0.45-0.5, were interpreted as the presence of relationship and a marginal relationship, respectively. Inter- and intraobserver agreements in the LPV in the bilateral ICA of the 30 patients were measured in 60 ICA segments and tested with the Bland and Altman plot method to estimate the validation of the semiautomatic measurement.

## 3. Results

**Figure 2:**
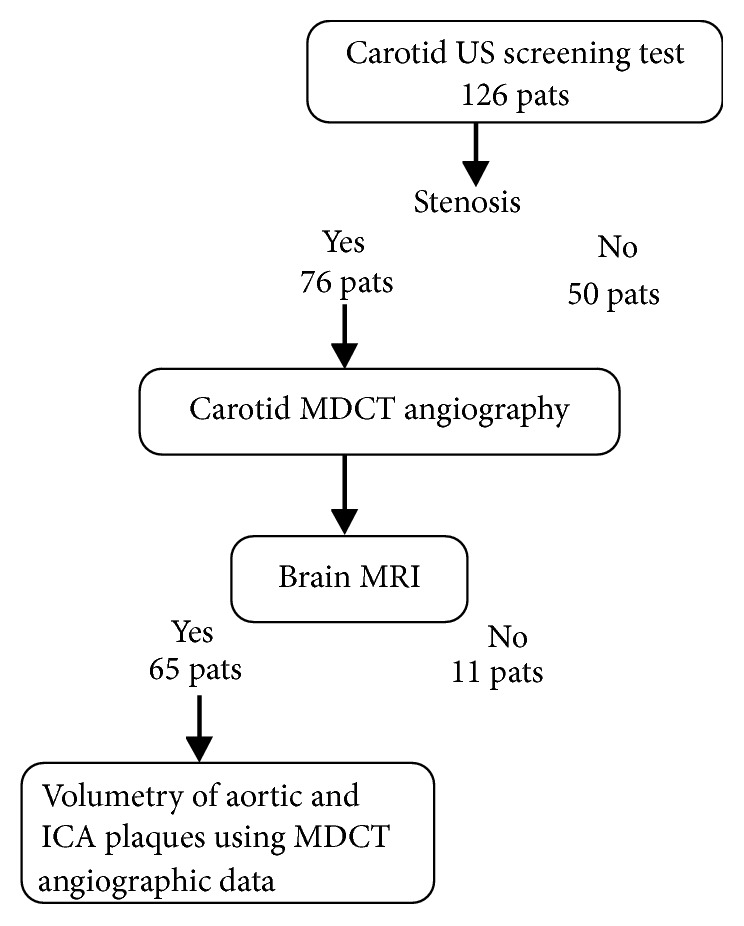
Flow chart showing number of patients who underwent volumetry of aortic and ICA plaques using MDCT angiographic data.

Of the 76 patients, 11 patients who did not undergo brain magnetic resonance imaging (MRI) were excluded from this study (Figure 2). Finally, the population of this study consisted of 65 patients between the ages of 50 and 87 years. Of the 65 patients, 28 patients younger than 70 years old were assigned to group 1 and 37 who were 70 years of age or older were assigned to group 2. Between the two patient groups, there were no significant differences in frequency of number of men, cerebral infarction, hyperlipidemia, and >30% ICA stenosis and the mean percentage of ICA stenosis ratio as well as mean diameters of the largest plaque in ICA (Table 1). Group 1 patients had significantly smaller mean TUPV in each aortic arch and ICA and LPV in ICA than group 2 patients (Table 2). There were no significant differences in the LPV-to-TUPV ratio in each aortic arch and ICA between those patient groups. The correlation coefficients between aortic plaques and ICA plaques were as follows. In group 1 patients, they were −0.280 in aortic LPV to ICA-LPV, −0.260 in aortic LPV to ICA-TUPV, −0.353 in aortic TUPV to ICA-LPV, and −0.425 in aortic TUPV to ICA-TUPV. In group 2 patients, aortic TUPV had a correlation with ICA-TUPV (r = 0.524, p = 0.001) and a borderline correlation with ICA-LPV (r = 0.483, p = 0.013) although aortic LPV had no correlations with ICA-LPV or TUPV (r = 0.337, 0.363, respectively).

**Figure 3:**
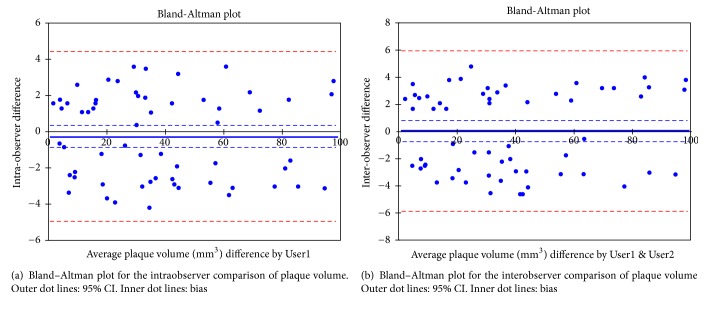
Bland–Altman plot for plaque volumes in 60 internal carotid arteries.

Table 3 shows coefficients of each aortic and ICA plaque volume, with the demographics, ICA stenosis, and plaque thickness. In group 1 patients, the aortic LPV had correlations with ICA percent stenosis ratio (r = 0.583, p = 0.001) and >30% stenosis in ICA (r = 0.714, p <0.001) and a marginal correlation with hyperlipidemia (r = 0.478, p = 0.011). The aortic TUPV in group 1 patients correlated with the numbers of men (r = 0.584, p = 0.001) and a borderline correlation with hyperlipidemia (r = 0.469, p = 0.001). However, cerebral infarction had no correlations with any measures in the aorta and ICA. There were no correlations with percentage of ICA stenosis ratio or >30% stenosis in the ICA with ICA plaque volumes. A correlation of ICA plaque volumes was limited to ICA-TUPV in group 2 with plaque thickness (r = 0.565, p < 0.001). Bland-Altman analysis indicated agreement between the two measurements of the plaque volume for intraobserver variation (r = 0.996, bias; −0.254 ± 2392, 95%CI: −0.872, 0.363; p = 0.413, Figure 3(a)) and interobserver variation (r = 0.994, bias; −0.047± 3.01, 95% CI: −0.731, 0.825; p = 0.904, Figure 3(b)).

## 4. Discussion

Atherosclerosis plaque depicted by MDCT can be divided into low-attenuation plaque, uncalcified soft-tissue attenuation plaque, and calcified plaque. A significant decrease in CT attenuation values was observed with increasing vulnerable plaque lipid, the amount of which was directly related to the content of lipid or hematoma and inversely related to the amount of fibrous tissue [6]. Low-attenuation values <30 HU inside the aortic and ICA lumen on CPR, using MDCT angiographic data, might reflect a large lipid core, necrosis, or hematoma. We applied a semiautomatic volumetric analysis of plaques with attenuation-dependent color codes, using CPR of MDCT angiographic data. Without inter- and intraobserver variabilities, the algorithm can provide accurate and quick measurement of attenuation of each ICA plaque, as shown in this study and the thoracic aortic plaques in our previous study [8].

The clinical significance of aortic plaque in association with other atherosclerotic disorders has been discussed. A significantly increased risk for all vascular events was observed in patients who had uncalcified aortic plaques greater than 4 mm in thickness, ulcerated plaques, or lipid-laden plaques. Cerebral infarction caused by plaques in the aortic arch is well known, and retrograde flow from the descending aorta has been found to be frequent in patients with determined or cryptogenic stroke [11]. In a large number of autopsy series, ulcerated plaques in the aortic arch were reported to be observed in 15% of patients with neurologic disease and suggested to play a part in causing cerebral infarction, especially in patients in whom cerebral infarction had no known cause [12].

There is literature about the correlation of aortic plaques and ICA plaques, although plaques in the carotid artery have been suggested to be correlated with aortic plaques because the demographic characteristics of patients with atherosclerotic lesions in the aortic arch are similar to those of patients with carotid plaques [4]. However, the severity of atherosclerosis in the cervical arteries was reported to be less than that in the aorta [12]. Atherosclerotic plaque should be evaluated after considering the patient's age because atherosclerosis plaques in the general population increase in prevalence with age, especially in patients who are older than 65 to 70 years. According to the ultrasound study of the ICA in the general population, plaques were observed in each, 3.0% and 8.3%, of the populations aged 50-69 years old and over 70 years old, respectively [13]. In this study of patients with ICA stenosis, the aortic TUPV correlated with TUPV in the ICA of patients older than 70 years. However, the plaque volumes in the aortic arch were not correlated with those in the ICA in the patients younger than 70 years old, and there was no significant difference in the ratio of LPV to TUPV in each age group.

The relationship of atherosclerotic plaques has also been investigated in relation to gender and some disorders. Male gender is one factor that is associated with aortic TUPV in this study and with ICA in the general population [13]. We have also reported that increasing LPV in the arch may be a significant factor associated with the development of severe atherosclerosis underlying abdominal aortic aneurysm, severe coronary arterial disease, and long-term hypertension [8]. Khoury et al. reported that each case of aortic plaques and ICA plaques is a predictor of coronary arterial disease to more or less extent, respectively [3, 14]. They also reported that evidence of aortic plaques in patients younger than 65 years old had a higher specificity and sensitivity for the coronary arterial disease [14]. In patients younger than 70 years old in our study, both LPV and TUPV in the aortic arch had marginal correlations with hyperlipidemia. In the general population, ICA plaque is a significant factor associated with the development of hypertension and hyperlipidemia [13]. In patients with brain infarction, the risk associated with aortic plaque thickness is markedly increased by the absence of plaque calcifications [4], although we did not find any association between aortic and ICA plaques and cerebral infarction in this study. Cerebral vascular disease caused by plaques in the aortic arch is suggested and retrograde flow from the descending aorta has been found to be frequent in patients with determined or cryptogenic stroke [11].

Regarding ICA stenosis, increasing LPV in the aortic arch is suggested to be a factor associated with the development of low-grade stenosis in the ICA. Curiously, we did not find any association between the plaques in the ICA and ICA stenosis by NASCET method, which has been used most commonly. One of the reasons for the discrepancy is that the NASCET criteria do not directly indicate ICA atherosclerosis because they do not include plaque measurements. The NASCET method underestimates the degree of stenosis if compared to the European Carotid Surgery Trial criteria which use the ratio between the residual luminal surface at the stenosis (inner-to-inner lumen) and the total surface (outer-to-outer), and including plaque measurement can indicate the degree of atherosclerosis [15].

There are some limitations to this study. First, there is no histological confirmation of the low-attenuation plaque in this retrospective study. Second, this study was conducted in a selected high-risk population whose sample size was not large.

Both the aortic arch and ICA plaque volumes can be measured clinically. The increasing aortic LPV may be a significant factor associated with the development of ICA stenosis in patients younger than 70 years old.

## Figures and Tables

**Table 1:** Comparisons of demographics, ICA stenosis, and plaque thickness in ICAs between group 1 patients and group 2 patients.

	Group 1 patients	Group 2 patients	p-Value
	< 70 years old	≥ 70 years old	
	(n =28)	(n=37)	
Mean age ± sd [years]	63.6 ± 5.8	75.8 ±3 .5	< 0.001
Number of men/number of women	14/14	19/18	0.931
No. (%) of cerebral infarctions	7 (25.0)	16 (43.2)	0.128
No. (%) of hyperlipidemia cases	12 (42.8)	18 (48.7)	0.712
Mean % ICA stenosis ± sd	33.4 ± 25.6	35.0 ± 20.0	0.596
No. (%) of >30% of ICA stenosis cases	13 (46.4)	19 (51.4)	0.694
Mean diameter of the largest plaques (mm) ± sd	2.41 ± 1.41	2.98 ± 1.07	0.056

sd: standard deviation.

**Table 2:** Comparisons of plaque volumes in aortic arch and ICA between 28 group 1 patients and 37 group 2 patients.

Volumes (mm^3^) of uncalcified plaque	Group 1 patients	Group 2 patients	p-value
	< 70 years old	≥ 70 years old	
	(n =28)	(n=37)	
	Mean mm^3^ ± sd	Mean mm^3^ ± sd	
Aortic arch			
LPV	297.0 ± 266.4	447.6 ± 586.9	0.371
TUPV	2734.2 ± 1351.9	3996.7 ± 2450.0	0.002*∗*
LPV/TUPV ratio (%)	9.7 ± 5.9	9.1 ± 5.6	0.921
ICA			
LPV	24.9 ± 22.5	44.1 ± 28.4	0.003*∗*
TUPV	183.8 ± 126.9	361.5 ± 118.3	< 0.001*∗*
LPV/TUPV ratio (%)	12.2 ± 7.3	11.4 ± 5.9	0.984

LPV: low-attenuation plaque volume, TUPV: total uncalcified plaque volume, sd: standard deviation.

**Table 3:** Univariate correlations of each low attenuation and totally uncalcified plaque volume in the ICAs with that in the aortic arch.

	Age (years- old)	Aortic LPV	Aortic TUPV	ICA LPV	ICA TUPV
Men	< 70	0.354	0.584*∗*	−0.089	−0.089
	≧70	0.122	0.111	0.056	−0.046

Cerebral infarction	< 70	0.209	0.281	−0.431	−0.399
	≧70	−0.077	0.041	−0.194	−0.092

Hyperlipidemia	< 70	0.478*∗∗*	0.469*∗∗*	−0.319	−0.301
	≧70	0.152	0.294	0.132	0.172

% stenosis ratio in ICA	< 70	0.583*∗*	0.197	−0.294	−0.360
	≧70	0.102	−0.016	0.056	0.043

>30% stenosis In ICA	< 70	0.714*∗*	0.403	−0.250	−0.250
	≧70	0.018	−0.023	−0.041	−0.068

Plaque thickness in ICA	< 70	0.405	0.029	−0.226	−0.309
	≧70	0.224	0.383	0.411	0.565*∗*

LPV: low-attenuation plaque volume, TUPV: total uncalcified plaque volume, *∗*: presence of correlation, and *∗∗*: marginal correlation.

## Data Availability

The data used to support the findings of this study are available from the corresponding author upon request.

## Abbreviations

ICA: Internal carotid artery
TEE: Transesophageal echocardiography
CT: Computed tomography
MDCT: Multidetector computed tomography
HU: Hounsfield units
CPR: Curved planar reformations
MPR: Multiplanar reconstruction
MIP: Maximum intensity projection
LPV: Low-attenuation plaque volume
TUPV: Total uncalcified plaque volume
NASCET: The North American Symptomatic Carotid Endarterectomy Trial.
